# Non-Interventional Monitoring on Antibiotic Consumption in a Critical Care Setting: A Three-Year Comparative Analysis

**DOI:** 10.3390/healthcare13151790

**Published:** 2025-07-23

**Authors:** Emanuela Santoro, Michela Russo, Roberta Manente, Valentina Schettino, Giuseppina Moccia, Vincenzo Andretta, Valentina Cerrone, Mario Capunzo, Giovanni Boccia

**Affiliations:** 1 Department of Medicine, Surgery and Dentistry “Scuola Medica Salernitana”, University of Salerno, 84081 Salerno, Italy; rmichela612@gmail.com (M.R.); vaschettino@unisa.it (V.S.); gmoccia@unisa.it (G.M.); vandretta@unisa.it (V.A.); mcapunzo@unisa.it (M.C.); gboccia@unisa.it (G.B.); 2 Clinical Pathology Unit, San Giovanni di Dio e Ruggi d’Aragona University Hospital, 84081 Salerno, Italy; manente392@gmail.com; 3 Clinical Oncology Unit, San Giovanni di Dio e Ruggi d’Aragona University Hospital, 84081 Salerno, Italy; valentina.cerrone@sangiovannieruggi.it; 4 DAI Department of Health Hygiene and Evaluative Medicine, A.O.U. San Giovanni di Dio e Ruggi d’Aragona, 84131 Salerno, Italy; 5 U.O.C. Hospital and Epidemiological Hygiene, A.O.U. San Giovanni di Dio e Ruggi d’Aragona, 84131 Salerno, Italy

**Keywords:** antimicrobial resistance, antibiotic consumption, intensive care unit

## Abstract

**Background/Objectives**: Hospitals are environments where care-related infections (HAIs) can occur, including those caused by resistant microorganisms. In addition, inappropriate use of antibiotics contributes to the development of antimicrobial resistance (AMR), a serious public health challenge. As part of the “Choosing Wisely—Italy” initiative, this study complements a previous publication on hand hygiene compliance in an intensive care unit (ICU) by analyzing antibiotic consumption over the same period and comparing it with the previous two years. **Methods**: A nine-month observational study was carried out from January to September 2018 in the ICU of a university hospital in Salerno province. Antibiotic order forms from the observation period were compared with those from the same months in 2016 and 2017. Glove consumption and costs were also analyzed over the three-year period. Statistical analysis was performed using ORIGIN* and EXCEL* software. **Results**: Overall antibiotic consumption during the observational period aligned with national averages reported in the National Plan to Combat Antimicrobial Resistance (PNCAR). **Conclusions**: These findings suggest that the presence of regular external monitoring may positively influence antibiotic use and hygiene behavior. Further research is needed to assess the long-term impact of observational interventions on clinical practice and AMR containment.

## 1. Introduction

Antibiotic resistance represents one of the most serious global threats in public health in the 21st century. The efficacy of antibiotics, a fundamental pillar of modern medicine, is being challenged by the increasing prevalence of resistant microorganisms, which undermine the ability to effectively treat common infections, increase the length of hospital stays, healthcare costs and mortality rates. The World Health Organization (WHO) has recognized antimicrobial resistance as a priority health emergency, calling for a coordinated, multisectoral global response [1]. The spread of antibiotic resistance is the result of various factors, including inappropriate and misleading use of antimicrobials in human and veterinary medicine (both in hospital and community settings), poor hygiene practices, inadequate infection control in hospitals, and the paucity of new antimicrobial agents in development [2]. According to the ECDC, there are more than 35,000 deaths in Europe each year that can be attributed to infections caused by antibiotic-resistant bacteria. This figure reflects the growing scale of the phenomenon, even in high-income countries with advanced healthcare systems [3]. The hospital is a place of care, but it can also become the place where one becomes ill from a hospital care-related infection (HAI). Combating HAI, and antimicrobial resistance in particular, is therefore a very important issue in public health and is the subject of the project “Choosing Wisely—Hospital Hygiene,” promoted by Anmdo (National Association of Hospital Management Physicians) and SItI (Italian Society of Hygiene, Preventive Medicine and Public Health) with the Gisio (Italian Hospital Hygiene Study Group) working group, as part of the Campaign “Doing More Doesn’t Mean Doing Better—Choosing Wisely Italy”. Based on available scientific evidence, five practices have been identified to be monitored with appropriate indicators, constituting a veritable vademecum for healthcare personnel. Do not use non-sterile disposable gloves instead of practicing good hand hygiene. An alcohol-based product should be used as the first choice for routine hand hygiene. Do not administer the antibiotic for perioperative prophylaxis less than 60 min before the surgical incision (with some exceptions); the ideal time is at the start of anesthesia. Do not administer antibiotics for perioperative prophylaxis more than 24 h after surgery. Antibiotic prophylaxis should be limited to the perioperative period. It is not justified to continue prophylaxis beyond the first 24 h postoperatively. It is important not to open the operating theatre doors during surgery, except when necessary for the passage of patients, staff and equipment [4]. The indications regarding the use of antibiotics mentioned above should fall within the concept of “rational use of drugs” defined by the WHO as the use of drugs appropriate to the clinical needs of patients, in the correct dose and for an adequate time. This represents a guiding principle for improving the effectiveness of treatments and counteracting phenomena such as antibiotic resistance [5].

It is important to document any positivity for alert microorganisms in the patient’s health records (discharge letter or transfer document to another facility). Assuming that good hand hygiene practice is one of the crucial points for the containment of healthcare-related infections, it must be admitted that, unfortunately, the levels of adherence to this practice are still uneven across the territory. In Italy, studies conducted at the Policlinico Umberto I in Rome showed compliance levels with hand hygiene of 70.6% and 63.1% in intensive care, with peaks of poor adherence especially “before contact with the patient”. These results, obtained through direct observations in hospital departments and intensive care units, highlight the need for recurring training interventions and periodic audits to improve hygiene practices even in high-intensity care contexts [6]. A systematic review conducted by Lambe et al. (2019) [7] analyzed 61 international studies to estimate hand hygiene adherence in intensive care units, based on the World Health Organization’s “Five Moments”. The average compliance rate found was 59.6%, with significantly higher values in high-income countries (64.5%) than in low-income countries (9.1%) and marked differences based on professional role: doctors showed the lowest level of compliance (32.6%), followed by nurses (43.4%) and other healthcare workers (53.8%). Furthermore, the review highlighted that hand hygiene moments aimed at protecting the patient (before contact or an aseptic procedure) are the least respected, while those aimed at protecting the operator (after exposure to fluids or contact with the patient) show higher levels of compliance. These results underline the need for targeted strategies to improve adherence in critical contexts and for specific categories of operators, with particular attention to the motivational and training aspect linked to patient protection [7].

We addressed a small part of the problem by extrapolating it from the overall design, and we tracked the operational activities relating to glove use, hand washing and patient handling by the three categories of staff who come into contact with patients: physicians, nurses and healthcare workers. We also correlated these data with the antibiotic consumption in the ward over a period of about a year, comparing the data with previous years when the protocol had not been applied. Antibiotics are medicines used to treat or prevent bacterial infections. They can kill bacteria and/or prevent them from multiplying and spreading within the body and being transmitted to other people [8]. Several studies have highlighted that the effectiveness of antibiotics in treating infections is continually decreasing [9]. Unfortunately, this decline is not offset by the availability of new effective antibiotics, as it was in the past, and is largely associated with their abuse and misuse. These trends have had a significant impact on Italy, one of the European countries with the highest antibiotic consumption (24.5 DDD/1000 people per day), alongside other southern European countries [3]. This could be due to the higher average age of the population, the high incidence of hospitalization and the poor appropriateness of prescriptions. A noteworthy contribution to this phenomenon could be due to regional differences in healthcare organization and the late adoption of structured antibiotic stewardship programs. Finally, insufficient vaccination coverage in certain population groups may also have contributed to a higher incidence of infections and antimicrobial resistance [10]. The international scientific community therefore broadly agrees on the need to curb the phenomenon by reversing the trend towards the appropriate use of currently available antibiotics, bearing in mind that resistance can be reduced to increase sensitivity, although this will happen less rapidly than the advance of antibiotic resistance. Since the beginning of the era of antibiotic therapy, which began with the introduction of penicillin in the 1940s, there has been a significant reduction in the impact of infectious diseases among the main causes of death [11]. However, antibiotic resistance represents a very significant public health problem globally because of the high epidemiological impact on the population (increased morbidity and mortality) and the related social and economic burdens (loss of life and work days, prolonged hospital stays, and increased use of diagnostic procedures). According to a recent (2020) analysis by the World Health Organization (WHO) in many parts of the world, high prevalences of resistance have been recorded in bacteria that cause even common infections, such as pneumonia and urinary tract infections [1]. The most frequently used antibiotics were found to be piperacillin and enzyme inhibitors (13.3%), Ceftriaxone (10.3%), levofloxacin (8.4%), Cefazolin (7.6%), Amoxicillin and enzyme inhibitors (7, 6%), Meropenem (5.2%), ciprofloxacin (5.1%), Ampicillin and enzyme inhibitors (3.3%), Fluconazole (3.2%), Sulfamethoxazole and Trimethoprim (2.3%) [12]. The three most prescribed classes of antibiotics (ATC Level IV) in 2017 are, in descending order, penicillins associated with beta-lactamase inhibitors (J01CR), fluoroquinolones (J01MA), and Generation III cephalosporins (J01DD), which together account for about two-thirds of the total consumption in public health facilities nationwide. This high use is due, particularly in high-intensity departments such as intensive care units, to the empirical choice of broad-spectrum drugs to promptly cover potentially resistant pathogens before culture confirmation [13]. In 2017, per capita spending on systemic antibiotics (J01) increased by 4 percent nationwide compared with the previous year, with regions in the center recording higher values, compared with those in the north and south [14]. In this context, recent studies underline the effectiveness of structured interventions to contain the phenomenon of inappropriate use of broad-spectrum antibiotics while promoting infection control and rationalization of antibiotic therapy based on individual risk and local microbiological data. One of the approaches suggested in the literature that could prove particularly effective is the implementation of structured antimicrobial stewardship programs that involve the active involvement of a multidisciplinary team. For example, it is suggested to establish teams composed of infectious disease specialists, clinical pharmacists, intensivists, microbiologists, nurses, infection control specialists and hospital epidemiologists, who can provide updated data on local epidemiology through internal surveillance, at an early stage of empirical prescribing. These figures could work in synergy not only to monitor and optimize the use of antibiotics, but also to train healthcare personnel on preventive measures, develop personalized guidelines and provide continuous feedback on prescriptive appropriateness and microbiological data [15]. In light of the previous considerations, the study was carried out in an anesthesia/intensive care unit of a university hospital of the Campania region. The observed ward mainly receives critically ill patients with severe respiratory, septic and postoperative conditions, with a high risk of care-related infections, making the setting particularly sensitive to hygiene and antibiotic prescription dynamics. This paper serves as a complement to a previous publication by the same research team, which examined the hand hygiene compliance of healthcare workers in an intensive care unit of a university hospital as part of the aforementioned WHO project [16]. The present study analyzes and compares the consumption data of the major classes of antibiotics utilized in a hospital setting during the same period of observation of hygiene practices with the consumption of the two previous years.

## 2. Materials and Methods

Antibiotic order forms made in the intensive care unit under study were taken into account during the observation period, and the data were compared with the previous two years. During the observation period, the following variables were monitored in parallel: consumption of antibiotics (by ATC classes), use of gloves, use of hydroalcoholic solution for hand hygiene and number of prescriptions. Observations were conducted by personnel outside the ward’s operational team, in a non-participant mode, in order to reduce the observer effect. The professional categories involved in the monitored activities included physicians, nurses and socio-medical workers, with no significant changes in staff composition during the period under review. Antibiotics were divided into classes according to PNCAR guidelines and using AIFA statistics on the most widely used antibiotics in Italy over the five-year period 2013–2017. All antibiotic prescriptions issued in the intensive care unit during the observation periods were included in the analysis. The precise total number of prescriptions could not be retrieved retrospectively in a disaggregated format, as the antibiotic orders were recorded at the unit level rather than linked to individual patients or prescribers. However, aggregated monthly data on the units of antibiotics ordered were available and were fully included in the analysis without exclusions. This limitation reflects the retrospective nature of the data collection process and the administrative recording system in place at the time of the study. Analyses were carried out on the units of antibiotic ordered and then used in the ward in the two previous years and in the current year of observation, dividing the antibiotics into classes: the most widely used fluoroquinolones (ciprofloxacin and levofloxacin) on which there is now strict ministerial health surveillance following the development of new, unclear side effects. In addition, oxazolidinones, beta-lactams, and rifampicin were examined. In all these cases, we analyzed the use of antibiotics before and after the observation period, using the orders placed by the department as a parameter. For the sake of completeness, a final graph was made of all antibiotics only during the period in which the observations were made, and this parameter was related to both the use of gloves and the use of hydroalcoholic solution for hand washing. The reported data were analyzed using the *OriginPro 2018* (OriginLab Corporation, Northampton, MA, USA)* statistical system and *Microsoft Excel 2016* (Microsoft Corporation, Redmond, WA, USA)*, highlighting trend lines and percentage error.

## 3. Results and Discussion

The results presented here are those of a purely descriptive study. No statistical tests were carried out, which could be a limitation of our study. The study has a descriptive design and does not include the use of inferential statistical tests. This choice represents a recognized limitation, which may be overcome in future studies with methodologically more robust designs to robustly quantify the effect of systematic observation activities on antibiotic consumption and hygiene practices. The data were analyzed and represented using ORIGIN* and EXCEL* software, with trends and percentage deviations highlighted. Indeed, the aim was to provide a description of the consumption of antimicrobials in a ward that is highly susceptible to care-associated infections and to assess a possible conditioning of behavior due to operators conducting an observation for the parallel study on hand hygiene compliance of healthcare workers mentioned earlier in this text. The observational approach was inspired by the behavioral strategies described in the literature, in particular social control theory and the COM-B (Capability, Opportunity, Motivation—Behavior) model, which suggest that external monitoring can strengthen motivation for change. Although no structured educational intervention was provided in the present study, the constant presence of observers potentially increased the perception of accountability, fostering behavior more in line with recommendations [17]. The observation period for antibiotics lasted nine months in 2018 and was compared with data from the same months in 2016 and 2017. The analysis of these data provided significant evidence both in terms of prescribing trends and compliance with the indications of the National Plan to Combat Antimicrobial Resistance (PNCAR) promoted by the Italian Hygiene Society (SItI) [6]. The methodological choice of extending the observation over a wide time span proved appropriate, allowing not only a more robust assessment of therapeutic compliance and clinical response, but also a more accurate analysis of the costs associated with the purchase and use of antibiotics in hospital wards.

**Figure 1:**
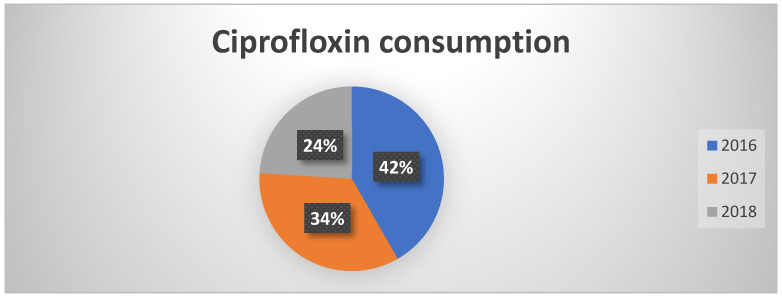
Percentages of ciprofloxacin consumption in the three years of observation.

Regarding the fluoroquinolones class, there has been a significant decrease in overall consumption, with the use of ciprofloxacin dropping sharply from 40% in 2016 to 24% in 2018 (Figure 1). This reduction aligns with the PNCAR’s recommendations, which emphasize surveillance and containment of antibiotics at high risk of resistance selection. Although there was a slight increase in levofloxacin use, the overall trend remains positive, indicating more rational prescribing.

**Figure 2:**
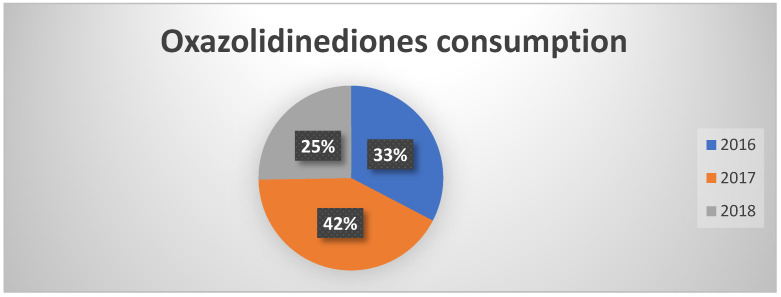
Percentages of oxazolidinedione consumption in the three years of observation.

Even for the class of oxazolidinones, which is subject to monitoring under PNCAR, a significant reduction has been observed already since 2017 (Figure 2). This figure confirms a growing attention of healthcare professionals towards a more conscious use of “monitored” antibiotics, with potentially positive effects on the prevention of the spread of resistant strains.

**Figure 3:**
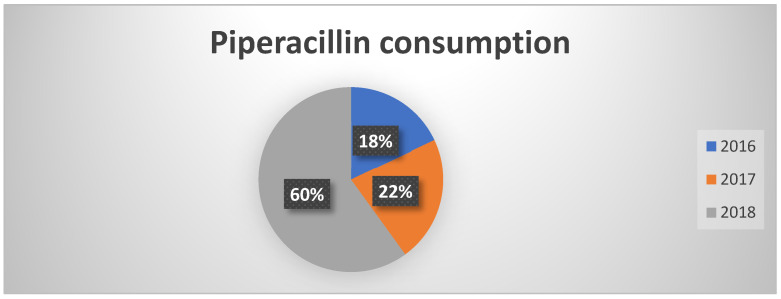
Percentages of piperacillin consumption in the three years of observation.

In contrast to previous data, piperacillin consumption showed an increase, consistent however with the national trend (Figure 3). It should be noted that, despite the increase, the trend in orders was irregular throughout the year, suggesting a targeted use at certain times and potentially related to specific infectious clinical pictures.

**Figure 4:**
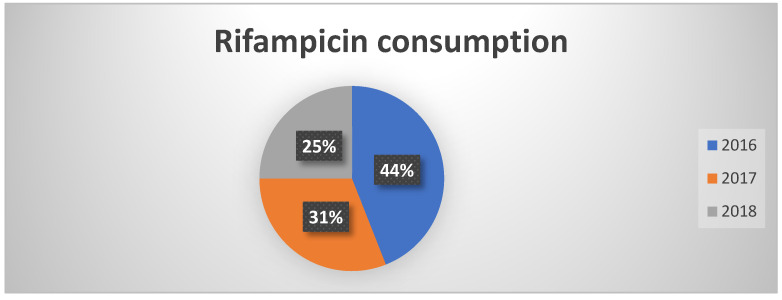
Percentages of rifampicin consumption in the three years of observation.

Consumption of rifampicin decreased from 44% in 2016 to 25% in 2018 (Figure 4). This reduction was not directly attributable to PNCAR, but rather to more effective management practices and improved hygiene conditions. This result highlights the vital role of infection prevention and control measures in reducing antibiotic use.

There is a significant reduction in the number of units ordered and consumed in the department. Finally, a comparative analysis of the monthly trend in total antibiotic consumption and the level of adherence to hand hygiene reveals an inverse relationship: higher levels of non-adherence to hygiene practices are associated with increased antibiotic consumption, and vice versa. This finding supports the idea that targeted interventions aimed at changing the behavior of healthcare workers, particularly with regard to hand hygiene, could significantly reduce nosocomial infections and, consequently, the use of antibiotics. The rational use of antibiotics can also represent a valid tool for containing antimicrobial resistance. The main methods to promote appropriate use include targeted prescribing based on microbiological criteria and updated guidelines; limiting the duration of therapy to the minimum effective level; the active surveillance of antibiotic therapy as well as antimicrobial stewardship programs aimed at guiding and monitoring prescribing practices in hospital departments, particularly in intensive care ones [18]. In summary, the results suggest that combined surveillance, education and hygiene promotion interventions, supported by national guidelines such as PNCAR, can effectively contribute to the containment of antimicrobial resistance, promoting a more appropriate use of antibiotic therapies and reducing the economic impact on healthcare.

## 4. Conclusions

The observation on the use of antibiotics lasted for a period of nine months. During this time period, the consumption of antibiotics in the department followed the trend of the national average, as envisaged by the National Plan to Combat Antimicrobial Resistance (PNCAR). However, we believe that the reduction observed is not attributable solely to compliance with national guidelines, but also to constant and systematic observation of the daily practices carried out in the department. This continuous presence, although without direct intervention, has favored a progressive awareness on the part of healthcare workers regarding the importance of hand hygiene and cleaning procedures. This awareness has translated, over time, into a measurable improvement in compliance, as demonstrated by the concomitant reduction in the use of antibiotics and gloves, and an increase in adherence to hand washing practices. Although the data suggest a positive effect of systematic monitoring on hygiene practices and the use of antibiotics, the results should be interpreted with caution in light of the absence of inferential statistical analysis, the lack of detailed patient classification and the possible influence of the observer on the behavior of the operators. The results obtained suggest a reflection that we believe is worthy of further scientific study: what could be the impact, in terms of effectiveness and quality of care, of a structured activity of external and regular observation of ward practices? The hypothesis that periodic meetings based on objective observations can significantly contribute to the improvement of care practices and the empowerment of operators opens up interesting scenarios for the future of clinical governance and the prevention of antibiotic resistance.

## Data Availability

The original contributions presented in this study are included in the article. Further inquiries can be directed to the corresponding author.
